# Virtual Reality for Distraction and Relaxation in a Pediatric Hospital Setting: An Interventional Study With a Mixed-Methods Design

**DOI:** 10.3389/fdgth.2022.866119

**Published:** 2022-05-31

**Authors:** Sylvie Bernaerts, Bert Bonroy, Jo Daems, Romy Sels, Dieter Struyf, Inge Gies, Wessel van de Veerdonk

**Affiliations:** ^1^Expertise Unit Psychology, Technology and Society, Thomas More University of Applied Sciences, Antwerp, Belgium; ^2^Mobilab & Care, Thomas More University of Applied Sciences, Geel, Belgium; ^3^Creative and Innovative Business, Thomas More University of Applied Sciences, Mechelen, Belgium; ^4^Department of Pediatrics, Universiteit Brussel (VUB), Universitair Ziekenhuis Brussel (UZ Brussel), Brussels, Belgium; ^5^Thomas More University of Applied Sciences, Mechelen, Belgium

**Keywords:** virtual reality, pediatrics, relaxation, implementation, anxiety, stress, feasibility, acceptability

## Abstract

Accumulating evidence supports the use of virtual reality (VR) as an effective pain and anxiety management tool for pediatric patients during specific medical procedures in dedicated patient groups. However, VR is still not widely adopted in everyday clinical practice. Feasibility and acceptability measures of clinicians' experiences are often missing in studies, thereby omitting an important stakeholder in VR use in a clinical setting. Therefore, the aim of this mixed-methods study was to investigate the feasibility, acceptability, tolerability (primary outcomes), and preliminary effectiveness (secondary outcome) of Relaxation-VR in both pediatric patients aged 4–16 years and clinicians. Relaxation-VR is a VR application prototype aimed to provide distraction and relaxation for a variety of patient populations and procedures and is used to reduce anxiety, stress (tension) and pain for children in hospital. Multiple measures of acceptability, feasibility and tolerability, and pre-to-post changes in measures of pain, anxiety, stress and happiness were assessed in pediatric patients. At the end of the study, acceptability and feasibility of VR use was assessed in clinicians. Results indicate that VR use (in particular, the Relaxation-VR prototype) for both distraction and relaxation is acceptable, feasible and tolerable for a variety of pediatric patients aged 4–16 years, as assessed in both patients and clinicians, and can reduce anxiety, pain and tension (stress), and increase happiness in a hospital setting.

## Introduction

Virtual reality (VR) is a form of human-computer interaction technology that immerses an individual in a computer-generated environment. While earlier versions of VR have included the use of large screens or 3-D glasses (semi-immersive VR), nowadays, VR environments are mainly experienced through advanced head-mounted displays (also called headsets) with built-in motion tracking (immersive VR). The specific immersive nature of this technology has made VR an interesting distraction method in pediatric pain and anxiety management (research) for over 20 years. Seminal work by Hoffman et al. (1) assessing the effects of VR during wound care in two case studies of adolescent patients with burn wounds suggested that immersive VR is an effective pain management tool. Following studies assessing the effects of immersive VR for pain management during burn wound care corroborated these findings (2–5). In addition, in pediatric patients (immersive), VR has been shown to be effective for alleviating pain and anxiety during post-surgical physical therapy (6), venous port access procedures (7), intravenous procedures (8–12), dental treatment (13), and for pain management of vaso-occlusive pain episodes in patients with sickle cell disease (14). In general, accumulating evidence has supported the use of VR as an effective pain and anxiety management tool for pediatric patients during specific medical procedures in dedicated patient groups [for review, see (15–18)]. In particular, a recent meta-analysis reported that the use of VR was significantly more effective in reducing pain (14 studies) and anxiety (7 studies) than standard care, with large effect size (17). Despite this growing evidence base, VR is still not widely adopted in everyday clinical practice.

To improve translation from research to practice, more knowledge on implementation factors is needed. A group of 21 international VR experts (Virtual Reality Clinical Outcomes Research Experts—VR-CORE) reported recommendations for research methodology on using VR in healthcare and defined three phases of VR clinical study designs: VR1, VR2, and VR3 (19). VR1 studies result in VR content development through a human-centered design with input from end-users. Afterwards, the product should undergo initial assessment in the target population within a representative clinical setting. This type of study, VR2, is aimed at assessing feasibility, acceptability, tolerability, and initial clinical efficacy. Lastly, VR3 trials involve clinical validation of the VR product, by means of prospective, adequately powered, methodologically rigorous randomized, controlled trials. In line with these recommendations, evidence on the feasibility, acceptability and tolerability of a VR interventions is growing. However, when these outcomes have been assessed to date, the measures to do so varied largely. For example, tolerability of VR has often been assessed in patients, seen as simulator sickness (or VR sickness) is a known side effect of VR use. VR sickness is similar to motion sickness and is thus characterized by symptoms such as nausea, dizziness, headaches or blurry vision (20). Some studies have specifically assessed nausea with a separate question or scale (1, 3, 5, 21), whereas others have assessed simulator sickness with a specific questionnaire (10, 22). In general, VR seems to be well-tolerated by pediatric patients with studies reporting no nausea or other side effects, and only mild when present. Concerning feasibility, this concept has been assessed by measuring experienced fun during VR use (3, 23), ease of use and understanding (usability) (8, 21), the occurrence of technical issues and procedure time (8). Together, these studies have shown that VR use is feasible for patients with burn wounds during physical therapy (3, 23), for patients with sickle cell disease (14), and during pediatric hemophelia care for distraction during intravenous interventions (8). With respect to acceptability, researchers have assessed this concept with open-ended questions or by measuring their intention or willingness to use VR again and found that VR use was acceptable for pediatric patients (14, 21). Taken together, these studies have each shown that various VR applications for distracting pediatric patients (from pain procedures) are feasible, acceptable and tolerable, each for use in a specific patient sample or during a specific medical procedure. Nonetheless, implementation of VR outside of the laboratory is lagging, and costs, user's attitudes and safety considerations (among others) are known implementation challenges (24). Therefore, an application intended for multiple uses, namely distraction and relaxation, and usable in pediatric patients of various ages and with various medical conditions, might prove to be more cost-efficient. With this study, we thus aim to assess the feasibility, acceptability and tolerability of a VR relaxation application to distract pediatric patients during various medical procedures as well as to provide relaxation during a longer hospital stay. Based on aforementioned literature, we hypothesize that one VR relaxation application for use in a varied patient sample is also feasible, acceptable and tolerable. To the best of our knowledge, feasibility and acceptability measures of clinicians' experiences are often missing in the aforementioned studies, thereby omitting an important stakeholder in VR use in a clinical setting. Therefore, the aim of our study was to investigate the feasibility, acceptability, tolerability (primary outcomes), and preliminary effectiveness (secondary outcome) of Relaxation-VR both from a patient and clinician perspective, which is in line with the VR CORE VR2 trial recommendations (19). Note, however, that this study does not aim to assess moderators nor predictors of VR acceptability, feasibility and tolerability for pediatric patients in hospital.

Relaxation-VR is a VR application (prototype) aimed to provide distraction and relaxation for a variety of patient populations and procedures, that is used to reduce anxiety, stress (tension) and pain for children in hospital. Also, no specific medical procedure, treatment or patient population is chosen in order to increase the generalizability of the findings and, therefore, the potential usability in clinical practice.

## Materials and Methods

### General Study Design

This mixed-methods study was performed at the University Hospital Brussels (Jette, Belgium; further named as UZ Brussel) and the General Hospital Sint-Maarten (Mechelen, Belgium; further named as AZ Sint-Maarten) to assess the acceptability, feasibility, tolerability and preliminary effectiveness of Virtual Reality (VR) as a relaxation and distraction tool for children admitted to the hospital. To do so, we used the Relaxation-VR app, a VR application (prototype) aimed to provide distraction and relaxation for a variety of patient populations and procedures. Before and after one VR session with Relaxation-VR, multiple measures of acceptability, feasibility and tolerability were assessed in pediatric patients. In addition, pre-to-post changes in measures of pain, anxiety, stress and happiness were assessed in pediatric patients. At the end of the study, after using Relaxation-VR with multiple patients, acceptability and feasibility of VR use was assessed in clinicians. Written informed consent was obtained from all participants prior to the study. Consent forms and study design were approved by the Medical Ethics Committee of the University Hospital Brussels (EC-20202-305) as well as by the local ethical committee of the AZ Sint-Maarten in accordance to the Code of Ethics of the World Medical Association (Declaration of Helsinki). The trial was registered at ClinicalTrials.gov (NCT04666506).

### Participants

**Figure 1:**
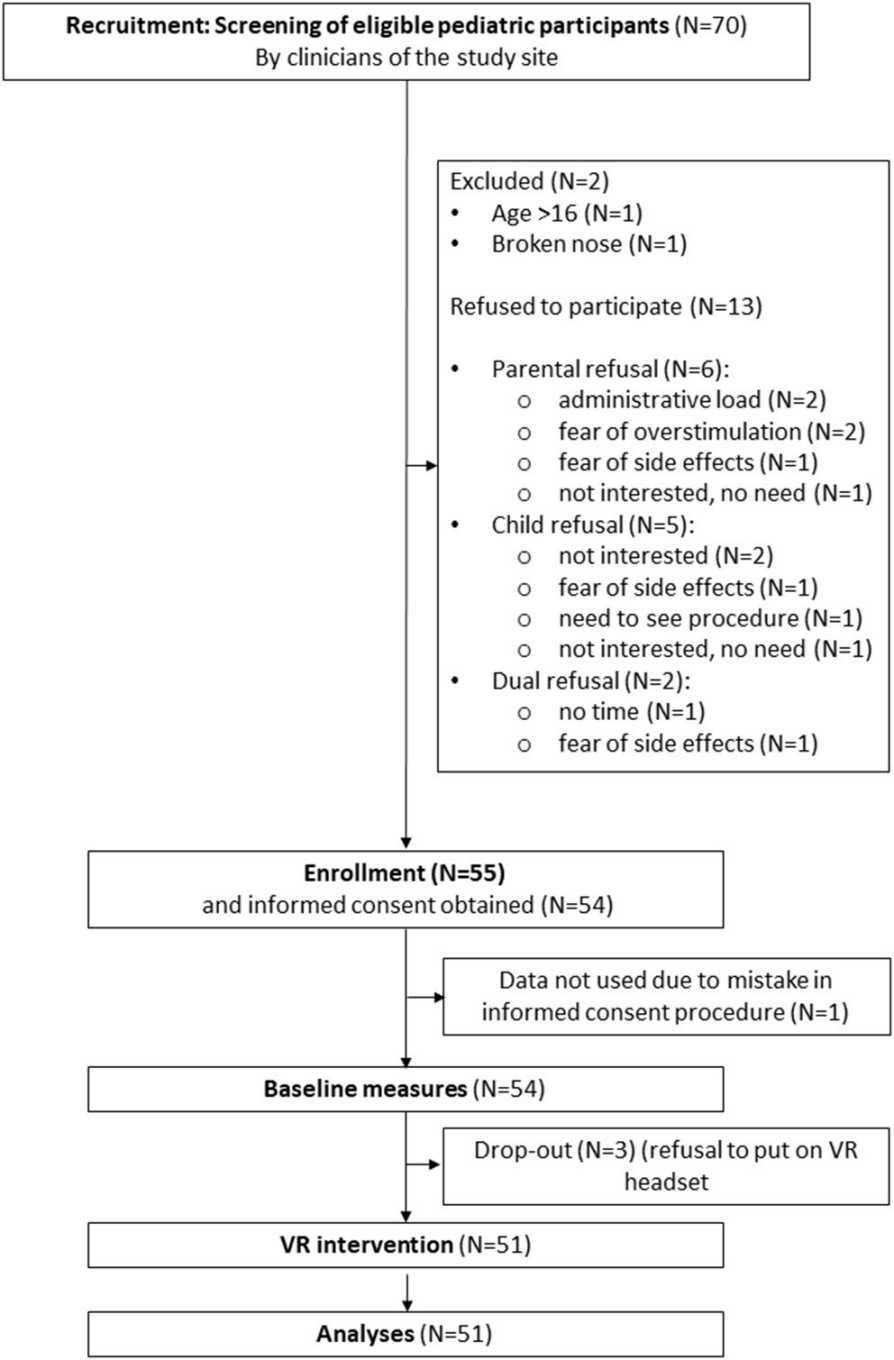
Flow chart.

Fifty-five pediatric in- and outpatients aged 4–16 years (*M* = 10.88, SD = 3.17, median = 11.00, minimum = 4, maximum = 16) were recruited between January and May 2021 from the UZ Brussel (*N* = 39) and AZ Sint-Maarten (*N* = 12) pediatric departments. Patients were included according to the following criteria: age between 4 and 16 years, normal or corrected-to-normal vision and hearing, in- or outpatient in one of the study sites and in need of relaxation or distraction before treatment of medical procedure, as assessed by the clinical staff. Exclusion was based on the following criteria: history of seizures, physical impairment that precludes VR intervention, need for medical procedures considered unsuitable in combination with the use of a VR headset, non-Dutch native patient or caregiver, or previous enrolment in the current study (during a previous hospital stay). Data from four participants were not included in the final analyses due to drop-out (*N* = 3, refusal to put on VR headset) or a mistake in the informed consent procedure (*N* = 1). Final analyses were therefore performed with data of 51 pediatric patients. Regarding clinicians, twelve women (11 nurses and 1 remedial educationalist) employed at the pediatric departments of UZ Brussel and AZ Sint-Maarten have participated in the study after informed consent was obtained. Demographic information of the study sample is described in Table 1 and the study flow chart is presented in Figure 1.

**Table 1:** Demographic information and baseline descriptives.

	**Pediatric patients (*N* = 51)**	Clinicians^*^(*N* = 12)
Gender	30 F/21 M	12 F/0 M
Age (*M*, SD)	10.88 (3.17)	30.08 (8.00)
Study site	39 B/12 SM	9 B/3 SM
**Prior knowledge**
No	26/51	
Yes	24/51	
Gaming	11/24	
Culture	8/24	
Owns VR headset	4/24	
Hospital	1/24	
Unknown	1/51	

*M, mean; SD, standard deviation; B, UZ Brussel; SM, AZ Sint-Maarten*.

*Culture-related prior knowledge of VR refers to experience with VR in musea and/or exhibits*.

* *11 nurses and 1 remedial educationalist*.

### Intervention

**Figure 2:**
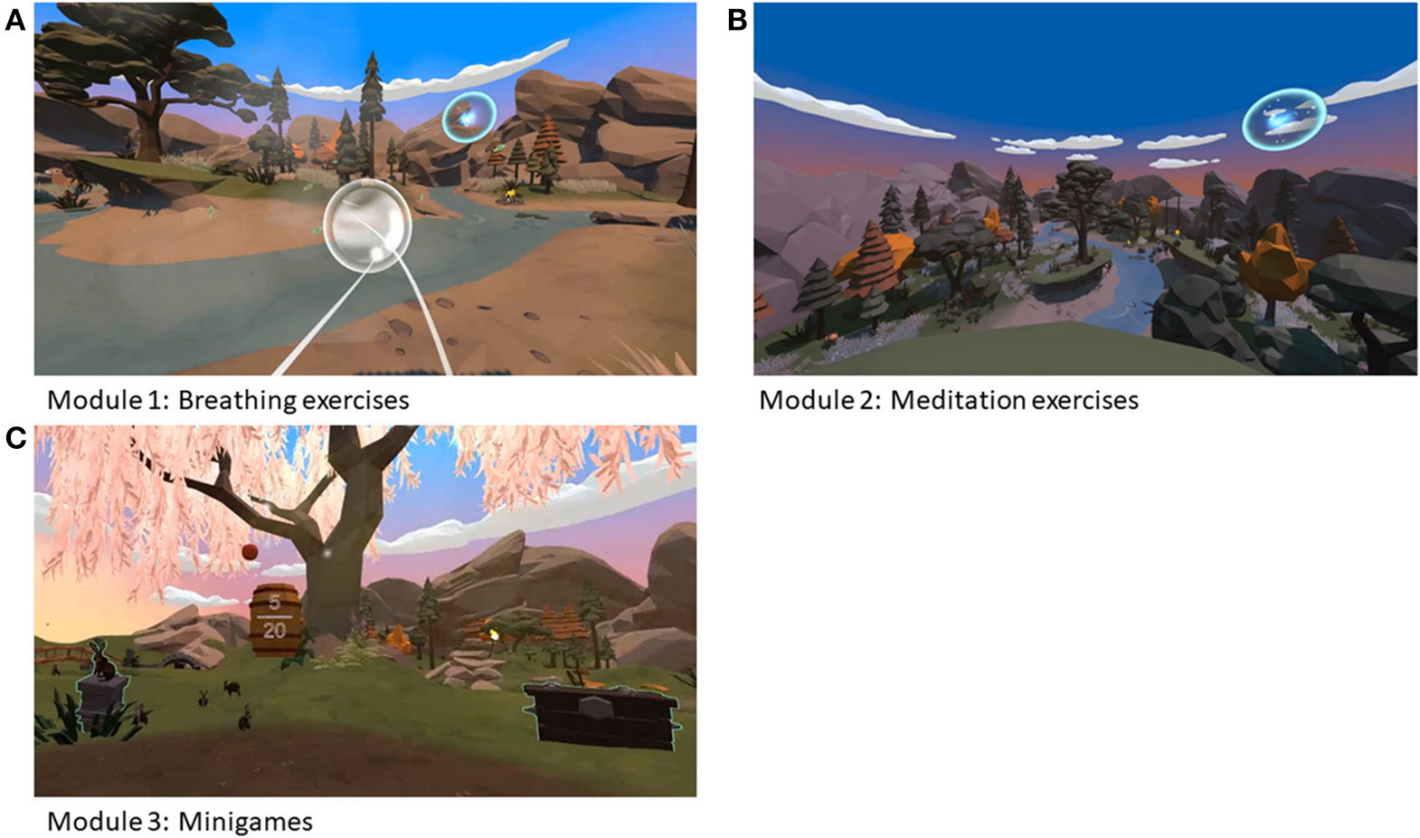
Images of Relaxation-VR modules 1 **(A)**, 2 **(B)**, and 3 **(C)**.

The Virtual Reality prototype application, named Relaxation-VR, is a VR application developed by Psylaris (25) aiming to reduce anxiety, stress and pain by distracting the patient in a relaxing environment. During the study, the Relaxation-VR app was made available *via* a commercially available Oculus Go VR headset (Meta, California, USA). By using the VR headset, participants are immersed in a novel, calming and distracting environment where they are asked to perform tasks that will help them to relax when distressed before and/or during a medical procedure or during their stay in hospital. The Relaxation-VR app consists of three modules or VR environments. The first module comprises breathing exercises; the second comprises meditation exercises (e.g., a body scan) and the third module presents a scene with different interactive animations and objects (i.e., catching falling apples, popping bubbles, and playing fetch with a dog) (Figure 2). Depending on their age, pediatric patients used either all three modules (aged 9–16) or only the third module (aged 4–8). This decision was based on prior discussions with the involved clinical staff and their experience in using relaxation exercises with (younger) patients. All participants received the VR intervention, which consisted of one VR session using the Relaxation-VR app.

### Procedure

After initial screening procedures by the clinical staff, pediatric patients and their parents were approached by the clinicians involved in the study at the pediatric departments of the study sites. Subsequently, interested patients and parents received the informed consent form (for parents and children aged 12 years and older) and assent form (for children aged 4–12 years old). Study procedures and legally and ethically required information were presented and explained in an informative video that was shown on a tablet in the hospital, any questions were answered by the local investigators. After the informed consent was obtained, participants filled in an online questionnaire *via* a tablet for demographic and background data collection and a paper questionnaire to collect baseline data on anxiety, pain, tension (stress) and happiness. If needed, parents were allowed to help their child to fill in the questionnaires. Participants then received the VR intervention. Participants were seated on a hospital bed or chair and also remained stationary in the virtual environment. The user was able to look around in the virtual environment by moving his/her head and was able to interact with virtual objects by staring at them for a few seconds, but did not move around in the different environments (modules). Modules 1 and 2 took ~6–7 min to complete, whereas participants were allowed to use module 3 for as long as needed. Afterwards, participants were asked to fill in the same paper questionnaire assessing anxiety, pain, tension (stress) during use of the VR intervention, as well as a self-composed paper and online questionnaire assessing acceptability, feasibility and tolerability of the intervention. Finally, at the end of the study (when the data of all pediatric participants was collected), the involved clinicians were asked to fill in an online questionnaire assessing acceptability and feasibility of the use of VR.

### Outcome Measures

Outcome measures were based on the recommendations of the Virtual Reality Clinical Outcomes Research Experts (VR-Core) for development and evaluation of therapeutic virtual reality, specifically on the recommendations for VR2 trials focusing on acceptability, feasibility, tolerability (primary outcomes) and initial clinical effectiveness (secondary outcome) (19). Data were collected with both a paper and online self-developed questionnaire including validated instruments and open-ended questions.

#### Acceptability

In the context of this study, acceptability refers to the patient's and clinician's willingness to use the Relaxation-VR app (19). In pediatric patients, acceptability was assessed by collecting data on patients' and their parents' willingness to enroll in the study and their reason for enrolling in the study (collected prior to using the Relaxation-VR app). After using the Relaxation-VR app, the patients were asked to rate their willingness to use the VR intervention again using a visual analog sale (VAS) to answer the question “To what extent would you use the Relaxation-VR app again?”. A score of 0 represented “I definitely want to use it again” and a score of 100 represented “I definitely do not want to use it again”. Pediatric participants were also asked to rate the extent to which they would advise others to use Relaxation-VR with a score of 0 representing “I definitely advise it to others” and 100 representing “I definitely do not advise it to others”. In addition, at the end of the study, clinicians' attitude toward Relaxation-VR was assessed with the Unified Theory of Acceptance and Use of Technology (UTAUT) questionnaire based on Ebert et al. (26) and the Unified Theory of Acceptance and Use of Technology model (27). The Dutch version of this qualitative 31-item questionnaire consists of the following scales: performance expectancy (4 items), effort expectancy (4 items), social influence (4 items), attitude toward technology (4 items), facilitating conditions (4 items), fear (4 items), intention to use (3 items), and self-efficacy (4 items) (28). Scores range from 1, representing “do not agree at all” to 5, representing “completely agree”. Higher scores reflect positive attitudes, except for the fear scale, for which higher scores reflect increased fear. Mean scores and their standard deviation are calculated per scale.

#### Feasibility

In the context of this study, feasibility refers to the degree to which the VR intervention (Relaxation-VR) can be successfully integrated in the hospitals' usual care (19). In pediatric patients, feasibility was assessed by collecting data on likeability and usability, similar to Dunn et al. (8), after using the Relaxation-VR app. To do so, pediatric patients were asked to use a VAS to answer “How easy was it for you to use Relaxation-VR (VR headset)?” with a score of 0 representing “really easy” and 100 representing “really difficult”. To assess usability, pediatric participants were asked to use a VAS to answer “How much fun was it for you to use Relaxation-VR?” with a score of 0 representing “really fun” and 100 representing “not fun at all”. In addition, descriptive data on the use of the VR intervention was also collected, for example: the aim of VR use (distraction or relaxation), the medical procedures during which VR was used, the occurrence of technical difficulties, suggested changes to the intervention and early removal of the VR headset. At the end of the study, feasibility was assessed in clinicians with the Client Satisfaction Questionnaire (CSQ-3) (29, 30) and the System Usability Scale (SUS) (31). In short, the CSQ-3 consists of 3 items, scored from 1 to 4, examining client satisfaction with the received service or intervention. The questionnaire generates a total score ranging from 3 to 12, with higher scores indicating higher satisfaction. The SUS consists of 10 items with 5 response options ranging from “strongly disagree” to “strongly agree”. Total scores for the SUS range from 0 to 100, with higher scores indicating better usability.

#### Tolerability

Tolerability refers to the evaluation of adverse events in pediatric patients, related to either hardware or software components (19). In pediatric participants, tolerability was assessed with the pediatric simulator sickness questionnaire (Peds SSQ) immediately after the use of Relaxation-VR. The Peds SSQ is a version of the Simulator Sickness Questionnaire (SSQ) (20) modified for pediatric use, as previously reported by Tychsen and Foeller (22). The questionnaire contains 13 questions comprising four symptom categories: eye strain (questions 1–4), head and neck discomfort (questions 5 and 6), fatigue (questions 7 and 8), and dizziness or nausea (questions 9 to 13). Participants are asked to indicate how much discomfort they experienced concerning a specific symptom on a numerical scale ranging from 0 (No) over 3 (A little bit) to 6 (A lot). The numbers 0, 3, and 6 are accompanied, respectively, by a happy, neutral or sad smiley face. In addition, adverse events were registered. Tolerability was only assessed in actual users, thus the patients.

#### Preliminary Clinical Effectiveness

In order to explore the potential clinical effects of the VR intervention on pain, anxiety, tension (stress) and happiness, the Revised Faces Pain Scale (FPS-R) was used, a VAS to measure anxiety and the Self-Assessment Manikin (SAM) to measure tension and happiness. The FPS-R scale is used to assess the intensity of a child's acute pain from the ages of four or five onwards (32). The scale presents six horizontally arranged cartoon faces with expressions linked to a numeric scale ranging from 0 to 10 with 0 representing “no pain” and 10 representing “very painful”. Participants were asked to circle the face that indicates how much pain they feel. To measure anxiety (VAS), participants were asked to answer the question “How anxious do you feel right now?” with a score of 0 representing “not anxious at all” and 100 representing “worst anxiety imaginable”. The Self-Assessment Manikin (SAM) is a non-verbal pictorial assessment technique used to measure the pleasure (sadness-happiness), arousal (tenseness-calmness), and dominance (mastery) associated with a person's affective reaction to a wide variety of stimuli (e.g., visual, auditory, and physical) (33). The measure consists of three single-item scales, of which each scale presents five figures (manikins) linked to a numerical scale ranging from 1 to 9, with 1 representing “very happy” and “very calm” and 9 representing “very sad” and “very tense”. Only two of these scales were used in this study, namely the pleasure scale and the arousal scale, to measure happiness and tension (stress), respectively, as the dominance scale was not relevant for this study.

### Data Analysis

For the acceptability, feasibility and tolerability measures, descriptive statistics are reported in Tables 2, 3. The answers to the open-ended questions were categorized and analyzed as descriptive data. For the FPS-R, VAS anxiety and SAM outcome measures, pre-to-post differences were assessed with separate paired samples *t*-tests. All statistics were executed with SPSS version 27 (IBM Corp., NY, USA). The significance level was set at *p* < 0.05. Cohen's d effect sizes are reported with 0.2, 0.5, and 0.8 indicating small, moderate and large effect sizes, respectively (34). Cronbach's alpha's were calculated for the UTAUT.

**Table 2:** Descriptive statistics of pediatric participants.

**Acceptability**	***N***	***M***	**SD**	**Med**
Use again	50	16.10	26.19	3.00
Recommend to others	50	11.84	15.76	4.00
**Feasibility**	***N***	***M***	**SD**	**Med**
Usability (ease of use)	49	15.31	22.22	4.00
Likeability (fun)	50	13.92	21.12	2.50
	***N***	**Yes (** ***N*** **)**	**No (** ***N*** **)**	**Unknown (** ***N*** **)**
Quit prematurely	51	10	33	8
Technical issues	51	5	38	8
Suggested changes	51	18	33	0
**Tolerability**	***N***	***M***	**SD**	
**Peds SSQ**
Eye	51	1.03	1.55	
Head/neck	51	0.76	1.43	
Fatigue	51	1.12	1.43	
Dizzy/motion sickness	51	0.77	1.39	

*N, number; M, mean; SD, standard deviation; Med, median*.

**Table 3:** Descriptive statistics of clinicians.

**Acceptability**	***N***	***M***	**SD**	**Cronbach's α**
**UTAUT**
Performance expectancy (4 items)	12	3.25	0.90	0.83
Effort expectancy (3 items)^*^	12	4.11	0.48	0.81
Attitude (4 items)	12	4.19	0.59	0.86
Social influence (4 items)	12	3.42	0.66	0.65
Fear (3 items)^*^	12	1.78	0.51	0.64
Intention to use (3 items)	12	4.11	0.88	0.77
Self-efficacy (4 items)	12	3.63	0.70	0.72
**Feasibility**	***N***	***M***	**SD**	**Med**
CSQ-3 (3 items)	12	9.42	1.88	9.5
SUS (10 items)	12	70.83	12.45	73.75

*CSQ-3, Client Satisfaction Questionnaire-3; SUS, System Usability Scale, Unified Theory of Acceptance and use of technology questionnaire*.

* *Note that the Effort Expectancy subscale and Fear subscale originally consisted of 4 items. Based on the poor internal consistencies of the 4-item subscales (Cronbach's α effort expectancy= 0.61; Cronbach's α fear= 0.22), one item from each scale was excluded from analysis. The Facilitating conditions subscale (4 items) has been eliminated from analyses, as the Cronbach's α was negative*.

## Results

### Acceptability

#### Patients

The majority of recruited patients (55/70) were willing to enroll in the study. Fifteen individuals [either the child (*N* = 7), a parent (*N* = 6) or both (*N* = 2)] refused to participate for reasons including fear of side effects (*N* = 3), willingness to see the procedure (*N* = 1), risk of overstimulating the child (*N* = 2), too much paperwork (*N* = 2), disinterest (*N* = 3), lack of time (*N* = 1), absence of need (*N* = 2), or an overlooked exclusion criterion (*N* = 1). Reasons for willingness to try the application and enroll in the study, range from a need for distraction or relaxation to boredom and curiosity. Future use of VR in a hospital context was favorably scored with a median score of 3.00 [interquartile range (IQR) = 22, *N* = 50, *M* = 16.10, SD = 26.19]. Also, most pediatric participants would recommend the use of VR to others with a median score of 4.00 (IQR = 19, *N* =5 0, *M* = 11.84, SD = 15.76). Acceptability data is reported in Table 2.

#### Clinicians

Table 3 provides an overview of UTAUT scores. Generally, clinicians showed a positive attitude toward using technology and the expected effort needed to use VR, as well as an intention to use VR. Notably, healthcare professionals were not fearful toward using technology.

### Feasibility

#### Patients

With respect to the specific use of the VR application, 30 out of 51 participants used the Relaxation-VR app as a relaxation tool during hospitalization and 19 participants used the application during a medical procedure (missing, *N* = 2), while 19 out of 51 participants used the app for periprocedural distraction: blood draw (*N* = 9), (veni)puncture (*N* = 3), tube placement (*N* = 2), lactose test (*N* = 1), wound care (*N* =1), circumcision (*N* = 1) and unknown procedure (*N* = 2). The majority of participants used all three modules (28/51), 15 out of 51 participants only used module 3 (minigames) (missing, *N* = 8). Most participants (33/51) used the VR application until the end, whereas 10 participants quit prematurely for reasons including discomfort (*N* = 2), disliking the application (*N* = 1), technical issues (*N* = 1), willingness to see the medical procedure being performed (*N* = 2), termination by nurse (*N* = 1), wanting to change the VR module (*N* = 1) (unknown reason: *N* = 2; missing data on premature termination: *N* = 8). Five out of 51 participants reported technical issues including start-up issues and low battery levels (missing, *N* = 8). Most participants (33/51) did not suggest any changes to the application, whereas 18 participants suggested changes regarding content (e.g., more games) or hardware (e.g., location of the start button). Ease of use of the Relaxation-VR application was favorably scored with a median score of 4.00 (IQR = 29, *N* = 49, *M* = 15.31, SD = 22.22). Also, likeability of the Relaxation-VR application was favorably scored with a median score of 2.50 (IQR = 25, *N* = 50, *M* = 13.92, SD = 21.12). Data concerning ease of use of two participants is missing as well as likeability data of one participant.

#### Clinicians

Participants reported a mean total CSQ-3 score of 9.24 (SD = 1.88, Cronbach's α = 0.91) (total scores range from 3 to 12 with higher scores indicating higher satisfaction) and a mean total SUS score of 70.83 (SD = 12.45, Cronbach's α = 0.76), indicating good usability (Table 3).

### Tolerability

Concerning the Peds SSQ, reported simulator sickness was limited as mean item scores per subscale ranged between 0.76 and 1.12 (with 0 indicating no discomfort and 6 indicating a lot of discomfort) (Table 2). Note, however, that at least for some participants, the scores seemed to be linked to their pre-existing conditions, rather than symptoms related to VR use (e.g., indicating nausea when treated in hospital for a gastrointestinal condition). Another adverse event that was registered was bedwetting (not immediately) after using Relaxation-VR. Note, however, that clinical staff reported that bedwetting had also occurred earlier in this participant.

### Preliminary Clinical Effectiveness

**Figure 3:**
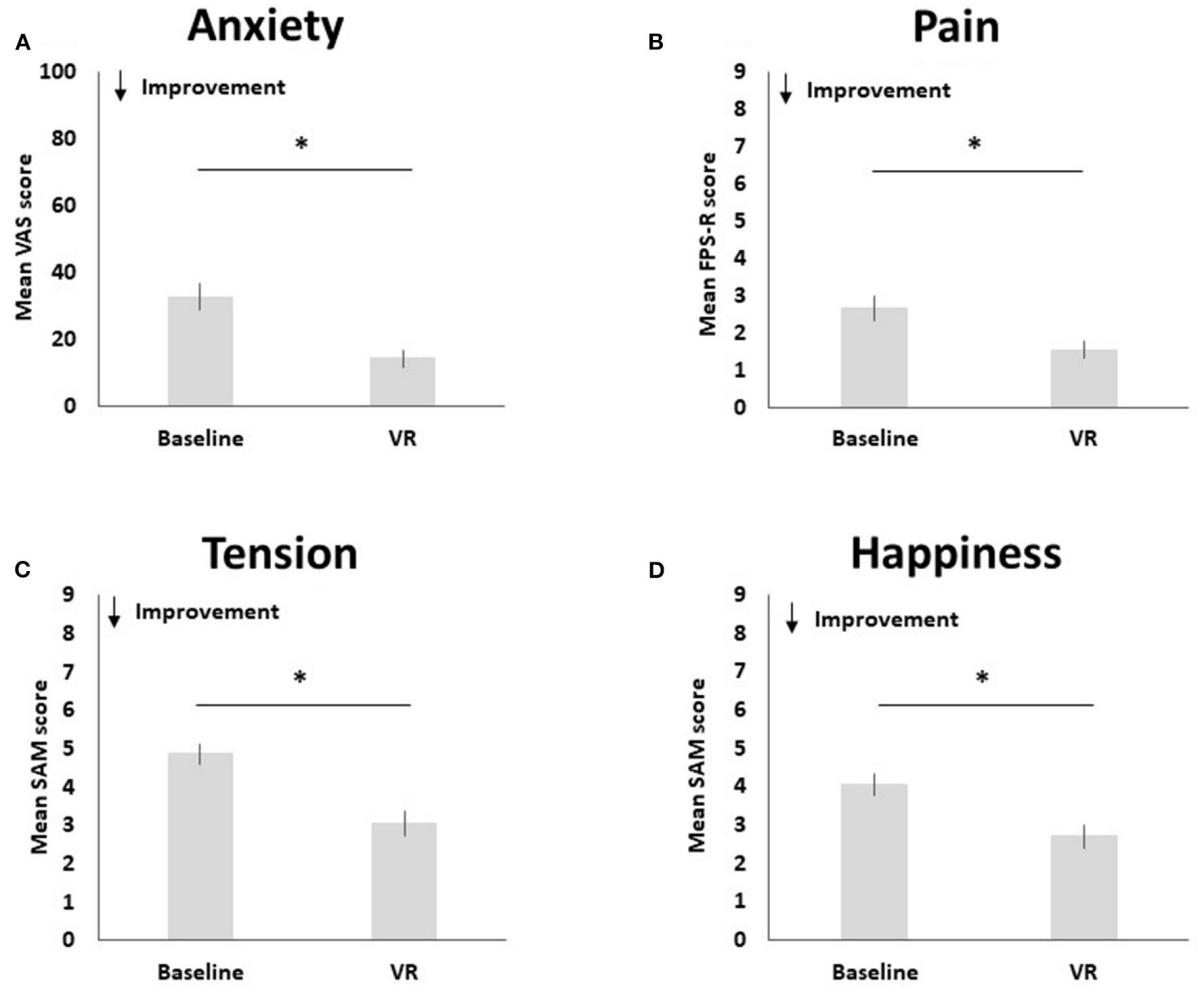
Pre-to-post changes in anxiety **(A)**, pain **(B)**, tension **(C)**, and happiness **(D)**. The * symbol indicates the significant group difference at the significance level of *p* < 0.001.

Pre-to-post changes in pain, anxiety, happiness and tension are visualized in Figure 3.

Compared to baseline (*M* = 2.65, SD = 2.37), pediatric participants reported less pain after using Relaxation-VR (*M* = 1.55, SD = 1.69), *t*(50) = 3.80, *p* < 0.001 (*d* = 0.53). Results indicate a significant decrease in reported anxiety after using Relaxation-VR (*M* = 14.34, SD = 18.48) as compared to baseline (*M* = 32.82, SD = 28.09), *t*(49) = 5.53, *p* < 0.001 (*d* = 0.78). Pediatric participants also reported significantly less tension after using Relaxation-VR (*M* = 3.06, SD = 2.17) as compared to baseline (*M* = 4.86, SD = 1.90), *t*(49) = 7.28, *p* < 0.001 (*d* = 1.03). Finally, results indicated that pediatric participants reported significantly higher levels of happiness after using Relaxation-VR (*M* = 2.71, SD = 2.07) as compared to baseline (*M* = 4.06, SD = 2.03), *t*(50) = 4.99, *p* < 0.001 (*d* = 0.70). Note that for the VAS and the SAM tension scale data of one participant are missing.

## Discussion

### Findings

This mixed-methods study was performed to assess the acceptability, feasibility, tolerability and preliminary effectiveness of virtual reality (VR) as both a relaxation and distraction tool for pediatric patients in a hospital setting. To do so, data from both patients and clinicians were collected. With respect to acceptability, most patients aged 4–16 years and/or their parents were willing to use VR with the aim of lowering their own (or their child's) anxiety or pain, out of mere curiosity, or due to boredom during a longer stay in hospital. Our findings indicate that pediatric patients accept the use of VR as a distraction and relaxation tool in a hospital. They wanted to use VR again during future hospital visits and would recommend it to others. These findings are in line with prior research indicating that participants (children, parents and nurses) would like to use VR distraction again during future procedures (8, 14, 21). In contrast, some patients recruited for this study and/or their parents were, however, unwilling to use VR for a variety of reasons, including fear of side effects. These patients and parents might benefit from clear and open communication by hospital staff about the potential side effects (or lack thereof). In line with prior research (1, 3, 5, 10, 14, 21), our study showed limited discomfort or side effects related to VR use. Most studies, however, assessed side effects or symptoms of VR sickness to a limited extent, and mostly focused solely on nausea, by means of a single question or graphic rating scale (1, 3, 5, 21, 35). Similar to our study, other studies assessing symptoms of VR sickness categorized into head ache, eye complaints and dizziness in addition to nausea, also revealed no or only mild symptoms (10, 14). Moreover, a recent study by Tychsen et al. (22) assessing the safety of VR use in young children (aged 4–10 years old) on visuomotor functions and posture showed that VR is tolerated without noteworthy effects on visuomotor functions or postural stability. Therefore, accumulating evidence seems to confirm the safety of VR use in a pediatric population. Nonetheless, the differences between these measures highlight the lack of a validated measure to assess VR sickness, side effects or safety of VR in children. Future research should, therefore, focus on creation of a measure of VR sickness in children. Our findings also revealed that clinicians accept the use of VR in clinical practice, reflected by a positive attitude toward using technology, the expected effort needed to use VR and intention to use it. However, comparable research on clinician attitudes toward or acceptability of VR use in hospital is lacking. Therefore, future studies should include acceptability measures to better understand why adoption of VR in practice is still limited.

Concerning feasibility, results revealed that the use of Relaxation-VR as a distraction and relaxation tool in pediatric clinical practice is feasible. Patients liked using VR and thought it was easy to use, which is in line with previous findings (3, 8, 23). Technical issues were limited and the majority of patients did not quit the application prematurely. Most participants did not suggest to make any changes to the application. However, the few suggested changes were similar to those found in prior research (14), including more games or environments and more interaction with the VR environment. As previously described, VR is mainly used and assessed as a distraction tool during specific procedures, whereas the potential of its use reaches further. Our results indicate that when given the instruction to clinicians to use the application as they see fit in their day to day practice, VR was more often used as a relaxation tool than as a distraction tool during medical procedures. Future research should therefore not only focus on pain and anxiety management through distraction, but also focus on incorporating and assessing evidence-based relaxation techniques for children in VR applications. The feasibility of using VR was additionally highlighted by the clinicians' reported satisfaction with and usability of the VR intervention. Prior research also indicates that clinicians (mostly nursing staff) are satisfied with VR use (36), but not necessarily more satisfied as compared to standard care (10). However, more implementation-focused research assessing feasibility, usability and acceptability from the perspective of clinicians is needed to gain insights in their perceptions and experiences with using VR.

With respect to (preliminary) clinical effectiveness, results revealed that pediatric patients reported less pain, anxiety and tension (stress) and higher happiness during VR use as compared to baseline measurements. Although different study designs have been adopted across the aforementioned studies, our findings are in line with prior research showing that the use of VR as a pain and/or anxiety management tool reduces periprocedural pain and anxiety levels in pediatric patients with various conditions and during various medical procedures (15–17). Evidence on the effects of periprocedural VR use on stress levels (tension) in pediatric patients is limited. Nonetheless, our findings corroborate previous findings by Piskorz et al. (9) indicating reduced stress levels during venipuncture in the VR intervention group as compared to the control group. A recent study investigating the effects of mindfulness-based VR (MBVR) in children with inflammatory bowel disease, showed that children felt more relaxed after using MBVR (similar to module 2 in the current study) and enjoyed its use (37), highlighting the potential of using VR as a relaxation tool for children in a hospital. The positive findings regarding higher reported happiness by the patients that VR use can improve a child's hospital experience.

Taken together, our results indicate that the use of VR is acceptable, feasible and tolerable, as assessed by both pediatric patients and clinicians, for providing both relaxation and distraction in a variety of pediatric patients as well as for periprocedural use and as a relaxation tool for children admitted to hospital for a longer period of time. The findings of this study confirm prior research indicating that VR is a relevant tool in a pediatric hospital setting and add to the evidence-base that VR use is also feasible and acceptable from clinicians' perspectives. Future research should, therefore, focus on potential barriers and facilitators to adoption of this innovation in clinical practice. Considering VR use was assessed in a varied population, during various procedures and for multiple purposes in one study, these findings also highlight that VR use in a hospital should not be limited to periprocedural distraction or specific patient groups, which might encourage adoption in clinical practice.

### Limitations

The presented findings should be considered in view of the following limitations. The study was unblinded and no control group was included. In addition, the nature of the participant-reported and clinician-reported outcomes may have introduced bias and subjectivity. The combination of these factors may have lead both patients and clinicians to provide more pleasing answers when completing the questionnaires and scales.

Concerning tolerability measures, we noticed that some pediatric participants had difficulties in discerning symptoms of VR use from symptoms related to their hospitalization. We therefore advise, researchers to include measures at multiple time points (i.e., at baseline and post-VR) to assess potential changes in VR sickness.

As the current study was not designed to assess the impact of potential moderators on the included outcomes, we cannot provide reliable information on the potential impact of age, gender, VR usage, type of medical procedures or other factors on study outcomes such as acceptability, feasibility and tolerability. Researchers are encouraged to design and conduct studies to explore the potential impact of these factors.

Note that this study was performed during the COVID-19 pandemic. All Belgian hospitals were subjected to national, regional and local regulations. These regulations may have influenced the number or type of patients available at the study sites. Regulations did not allow external researchers to visit the study sites to educate the clinical staff in study procedures, nor in VR use, so that the necessary education and support was provided online. Guidelines were developed to guide clinical staff in working with the technology and performing the research steps as planned.

## Conclusion

Study results indicate that VR use (in particular, the Relaxation-VR prototype) for both distraction and relaxation is acceptable, feasible and tolerable for a variety of pediatric patients aged 4–16 years, as assessed in both patients and clinicians, and can reduce anxiety, pain and tension (stress), and increase happiness in a hospital setting.

## Data Availability Statement

The raw data supporting the conclusions of this article will be made available by the authors, without undue reservation.

## Ethics Statement

The studies involving human participants were reviewed and approved by the Medical Ethics Committee of the University Hospital Brussels as well as by the Local Ethical Committee of the AZ Sint-Maarten. Written informed consent to participate in this study was provided by the participants' legal guardian/next of kin.

## Author Contributions

SB and IG have coordinated the study design. IG has coordinated data collection. SB has written the first and final versions of the paper. BB, JD, RS, DS, and WV have provided feedback on both the study design and the manuscript. All authors contributed to the article and approved the submitted version.

## Funding

This study has been conducted as part of the Immersive Care Project funded by the TETRA Program of the Flemish Government in Belgium (VLAIO, HBC.2019.2024).

## Conflict of Interest

The virtual reality prototype used in the study was developed by the Dutch company Psylaris. Psylaris was not involved in the study design, data collection or writing of this article. The authors declare that the research was conducted in the absence of any commercial or financial relationships that could be construed as a potential conflict of interest.

## Publisher's Note

All claims expressed in this article are solely those of the authors and do not necessarily represent those of their affiliated organizations, or those of the publisher, the editors and the reviewers. Any product that may be evaluated in this article, or claim that may be made by its manufacturer, is not guaranteed or endorsed by the publisher.
